# Time‐Dependent Efficacy of Lymphaticovenous Anastomosis in Secondary Lymphedema: A Rabbit Model Study

**DOI:** 10.1096/fj.202602801RR

**Published:** 2026-10-05

**Authors:** Ping Zhao, Wenzhao Liu, Mingqiang Liu, Jintao Yang, Sen Li, Xun Li

**Affiliations:** ^1^ Lanzhou University Lanzhou China; ^2^ Department of Hand and Foot Surgery The First Hospital of Lanzhou University Lanzhou China; ^3^ The First School of Clinical Medicine Lanzhou University Lanzhou P.R. China; ^4^ Department of General Surgery The First Hospital of Lanzhou University Lanzhou P.R. China; ^5^ Key Laboratory of Biotherapy and Regenerative Medicine of Gansu Province Lanzhou P.R. China; ^6^ Medical Engineering Center for Liver Repair and Regeneration of Gansu Province Lanzhou P.R. China; ^7^ Hepatopancreatobiliary Surgery Institute of Gansu Province Lanzhou P.R. China; ^8^ Clinical Research Center for General Surgery of Gansu Province Lanzhou P.R. China; ^9^ Innovation Center for Advanced Particle and Nuclear Medicine Technologies Lanzhou P.R. China

**Keywords:** animal model, lymphaticovenous anastomosis, secondary lymphedema, supermicrosurgery, therapeutic window, timing optimization

## Abstract

Secondary lymphedema remains a common complication after oncologic lymph node dissection. The optimal timing of lymphaticovenous anastomosis (LVA) is unclear due to a lack of time‐dependent large‐animal models. Twenty‐five New Zealand White rabbits underwent popliteal‐region surgery to induce secondary hind limb lymphedema. Animals received immediate LVA (*n* = 5) or delayed LVA at 4, 8, or 12 weeks after model induction (*n* = 5 per group); untreated lymphedema controls were included (*n* = 5). Outcomes included limb circumference/swelling rate, anastomotic patency by indocyanine green (ICG) fluorescence imaging, and histopathology/molecular markers (e.g., VEGF‐C, LYVE‐1). The model produced sustained hind limb swelling and molecular evidence of lymphangiogenic remodeling (e.g., increased VEGF‐C and LYVE‐1). Immediate LVA achieved 100% technical success with durable patency during follow‐up. Delayed LVA groups demonstrated patency rates of approximately 75%–80%. Immediate intervention resulted in the greatest long‐term reduction in swelling compared with delayed interventions. Mean lymphatic and venous diameters were approximately 0.6 and 0.8 mm, respectively, and mean operative time was ~55 min. This rabbit model provides a reproducible platform to evaluate time‐dependent LVA efficacy. Earlier intervention yielded superior functional outcomes, while delayed LVA remained feasible and maintained reasonable patency, supporting a clinically relevant therapeutic window.

## Graphical abstract

Secondary lymphedema was induced in New Zealand White rabbits by popliteal lymph node excision and afferent lymphatic vessel ligation, causing sustained hind limb swelling. LVA was performed immediately or after 4, 8, or 12 weeks. Patency was verified intraoperatively by microsuturing and postoperatively by indocyanine green (ICG) lymphangiography. Immediate LVA achieved 100% patency and the greatest reduction in limb circumference; delayed LVA showed lower patency (75%–80%) and incomplete resolution, indicating that earlier intervention yields superior outcomes in this model.

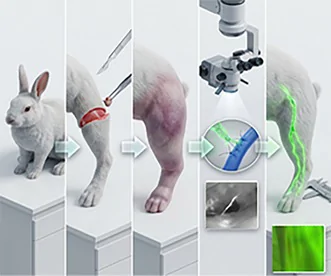

## 1 Introduction

Secondary lymphedema (SLD) is a debilitating complication following oncologic lymph node dissection, affecting cancer survivors across sexes and malignancy types. Clinical epidemiology indicates substantial burdens in breast and gynecological cancers (e.g., approximately 38% of breast cancer survivors and 29% of gynecological cancer patients) [1, 2]. The pathophysiology involves chronic inflammation and progressive fibrosis caused by impaired lymphatic drainage, leading to significant functional impairment and reduced quality of life [3].

Lymphaticovenous anastomosis (LVA) has emerged as the standard surgical treatment for early‐to‐moderate SLD [4, 5, 6]. Recent advances in supermicrosurgical techniques and intraoperative imaging have substantially enhanced procedural success rates [7, 8, 9]. Clinical observations demonstrate excellent outcomes in early‐stage lymphedema; however, efficacy decreases considerably once tissue fibrosis develops [10, 11], suggesting that intervention timing may be critical for optimal therapeutic outcomes.

A major limitation in mechanistic studies is the lack of suitable animal models that can simulate human SLD pathology with adequate temporal observation windows [12]. Existing models face significant constraints: small rodents suffer from anatomical disparities and spontaneous resolution that poorly reflect human pathophysiology, while larger animal models present ethical and cost limitations [13].

Our anatomical investigations revealed that New Zealand rabbits' popliteal region offers unique translational advantages. The lymphatic‐venous distance (0.8 ± 0.2 mm) closely approximates human anatomy (1.1 ± 0.3 mm), and lymphatic vessel diameter distribution (0.6–1.2 mm) parallels human extremities [14, 15]. This anatomical similarity, combined with accessible surgical approaches and reasonable maintenance costs, positions rabbits as an optimal experimental platform for LVA research. Secondary lymphedema after oncologic lymph node dissection is clinically relevant in both women and men, including post‐prostatectomy lymphedema following prostate cancer treatment [16]. To ensure that the model specifically represents secondary lymphedema arising after lymph node dissection, we used male rabbits. In female rabbits, estrous cycle–associated variations in systemic fluid balance and baseline soft‐tissue water content can lead to subtle edema fluctuations, which may obscure longitudinal quantification of LVA‐related changes. Therefore, using male animals improves reproducibility by minimizing potential hormonal confounding while maintaining the translational relevance of SLD pathophysiology.

The critical question of optimal intervention timing remains unresolved due to limited experimental evidence. Clinical observations suggest that early intervention yields superior outcomes, but the precise therapeutic window and mechanisms underlying time‐dependent efficacy have not been systematically investigated [17, 18]. Understanding these temporal relationships is essential for developing evidence‐based clinical protocols and exploring combination therapies.

We developed a novel time‐dependent rabbit model to systematically evaluate LVA efficacy across multiple postoperative intervals. This three‐stage experimental paradigm encompasses SLD induction, lymphatic remodeling phases, and sequential therapeutic interventions at 4, 8, and 12 weeks post‐induction. The model provides the first systematic platform for investigating the relationship between intervention timing and therapeutic outcomes, offering crucial translational foundations for optimizing clinical management protocols.

## 2 Materials and Methods

### 2.1 Animals and Experimental Design

A total of 25 male New Zealand white rabbits, weighing 2360 ± 168 g (range: 2000–2500 g), were included in this study. Male rabbits were selected to minimize confounding effects of estrous cycle‐related hormonal fluctuations on edema formation and inflammatory responses. All animals were housed in a specific pathogen‐free (SPF) environment with free access to food and water. The study protocol was approved by the Institutional Animal Care and Use Committee of the First Hospital of Lanzhou University (Approval No.: LDYYLL2026‐218). All surgical procedures were performed on the right hind limb. The sample size was determined based on previous similar experimental microvascular studies and the “resource equation” method, aiming to minimize animal use while ensuring sufficient statistical power to detect macroscopic and transcriptomic differences. The rabbits were randomly divided into five experimental groups (*n* = 5 per group):

1. Control Group (SLD): Rabbits underwent surgical induction of secondary lymphedema (SLD) without subsequent treatment.
2. Immediate LVA Group: Lymphaticovenous anastomosis (LVA) was performed immediately after the SLD model was established.
3. Delayed LVA Group: LVA was performed at 4 weeks post‐SLD induction.
4. Late LVA Group: LVA was performed at 8 weeks post‐SLD induction.
5. End‐stage LVA Group: LVA was performed at 12 weeks post‐SLD induction.

### 2.2 Anesthesia and Surgical Preparation

Chloral hydrate (300–500 mg/kg) was injected via the auricular marginal vein. A maintenance dose was administered as needed to ensure a consistent level of anesthesia. The experimental animal was positioned in dorsal recumbency, and the hair on both hind limbs was shaved. The surgical field was disinfected and draped following standard aseptic protocols.

### 2.3 Surgical Procedures

All microsurgical procedures were performed under a Zeiss Tivato 700 surgical microscope (Zeiss, Germany) using ultrafine microsurgical instruments (Linghu, China). Lymphatic and venous vessel diameters were measured with a Zeiss micro‐caliper.

#### 2.3.1 Indocyanine Green (ICG) Lymphography

To visualize the lymphatic drainage, 0.1 mL of indocyanine green (ICG, 2.5 mg/mL; Dandong Yichuang Pharmaceutical) was injected subcutaneously at three interdigital web spaces of the right hind limb. Lymphatic pathways were tracked in real‐time using a 4K fluorescence imaging system (Nuoyuan, China) (Figure S1).

#### 2.3.2 Establishment of the Secondary Lymphedema (SLD) Model

Following ICG mapping, a circumferential incision was made around the popliteal fossa. The popliteal lymph nodes and their afferent lymphatic vessels were identified under fluorescence guidance. The lymph nodes were completely excised, and the afferent lymphatic vessels were ligated with 6‐0 silk sutures to establish lymphatic obstruction. The skin incision was then closed with 4‐0 sutures (Figure S2). This combination of popliteal lymph node excision and circumferential skin incision was selected based on prior evidence that node excision alone does not reliably produce sustained macroscopic limb swelling in rabbits [19, 20], whereas the addition of circumferential incision ensures disruption of collateral lymphatic drainage pathways and reproducible edema formation.

#### 2.3.3 Lymphaticovenous Anastomosis (LVA)

In the LVA groups, after SLD model creation, the popliteal vein adjacent to the main lymphatic trunk was identified. An end‐to‐end or end‐to‐side anastomosis was performed between the proximal lymphatic segment and the distal venous segment using 11‐0 or 12‐0 nylon sutures. For delayed procedures (Delayed, Late, and End‐stage groups), the target vessels were re‐identified by locating suture markers placed during the initial surgery. Anastomotic patency was confirmed intraoperatively by observing ICG flow from the lymphatic vessel into the vein using fluorescence imaging (Figures 1 and 2).

**FIGURE 1:**
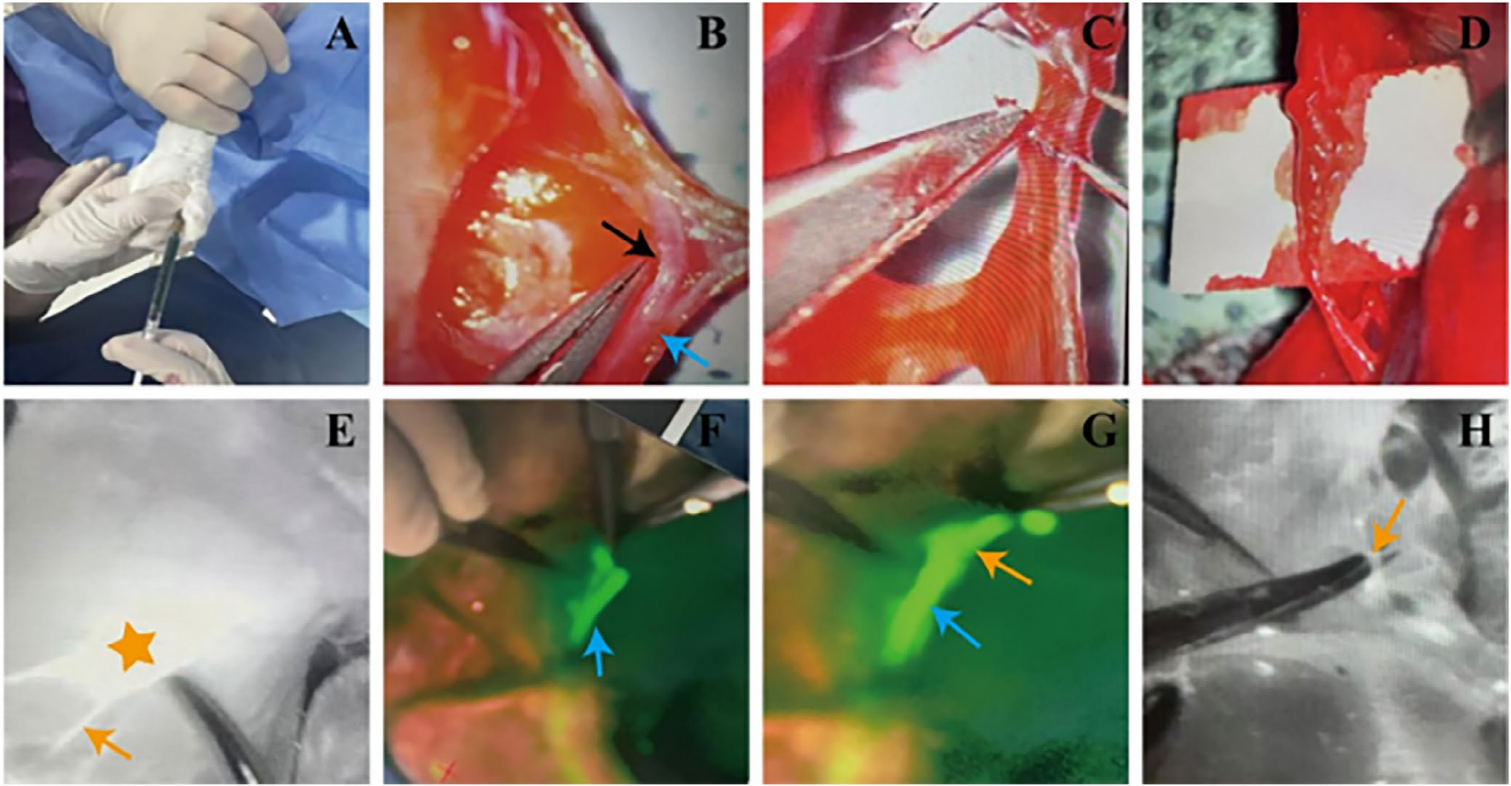
Immediate lymphaticovenous anastomosis (LVA) following model establishment. (A) Digital web injection of indocyanine green; (B) Identification of the vein and adjacent lymphatic vessels (black arrows point to the vein, blue arrows point to the lymphatic vessel); (C) Anastomosis of the lymphatic vessel and vein; (D) Patent lymphaticovenous anastomosis; (E) Pre‐lymph node excision fluorescence showing lymph nodes and target lymphatic vessels (orange star indicates lymph node, orange arrow indicates target lymphatic vessel); (F) Merged imaging shows lymphatic vessels and veins (blue arrows point to veins containing small amounts of indocyanine green, which are visible on merged imaging but not clearly visible on fluorescence imaging alone; blue arrows indicate lymphatic vessels); (G) Mixed fluorescence showing completed lymphaticovenous anastomosis (indocyanine green has completely disappeared from the vein at the position previously indicated by blue arrows, blue arrows indicate lymphatic vessels, orange arrows point to the anastomosis site); (H) Monochrome fluorescence showing patent lymphaticovenous anastomosis with a small amount of indocyanine green flowing into the proximal end of the vein.

**FIGURE 2:**
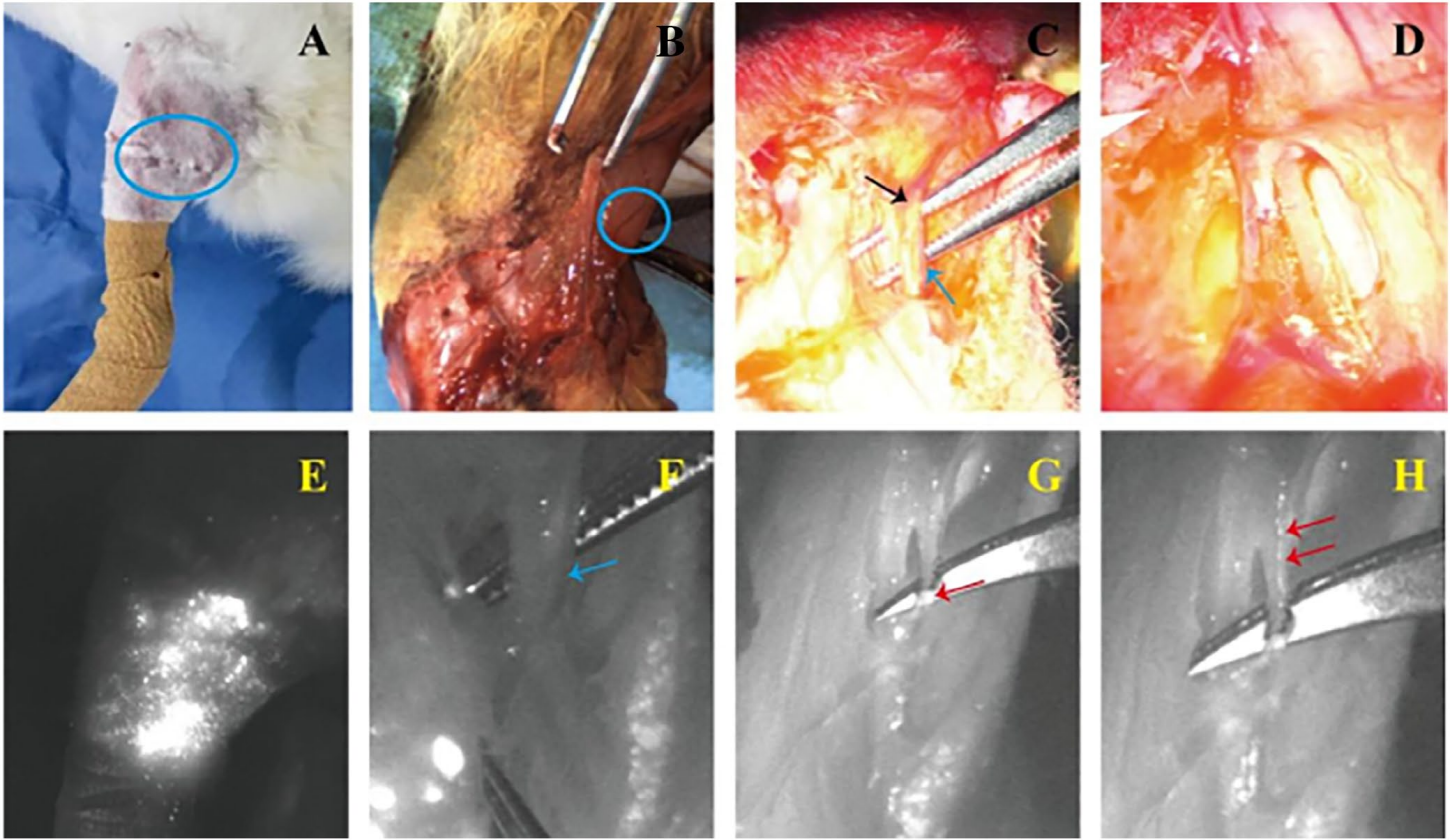
Delayed lymphatic‐venous anastomosis model. (A) Healed previous surgical incision visible within the blue circle; (B) Identifying the marking line (within blue circle); (C) Identifying veins and lymphatic vessels for anastomosis (black arrows indicate veins, blue arrows indicate lymphatic vessels); (D) Completed lymphatic‐venous anastomosis with confirmed patency; (E) Fluorescence imaging shows irregular spotty accumulation of indocyanine green in the distal limb, further confirming successful SLD model; (F) Fluorescence imaging shows no significant flow of indocyanine green within the lymphatic vessels (indicated by blue arrow); (G) No indocyanine green visible in the proximal vein at the completion of lymphatic‐venous anastomosis (orange arrows indicate the lymphatic‐venous anastomosis site); (H) Small amount of indocyanine green appears in the vein approximately 10 min after anastomosis completion (indicated by orange arrow).

### 2.4 Postoperative Evaluation

#### 2.4.1 Limb Circumference Measurement and Swelling Rate Calculation

To minimize bias, all postoperative limb circumference measurements and histological evaluations were performed by investigators who were blinded to the group allocations. Limb circumference was measured weekly at a marked position 5 cm above the ankle joint for both hind limbs. The swelling rate (SR) was calculated using the formula: SR (%) = [(Circumference of affected limb − Circumference of contralateral limb)/Circumference of contralateral limb] × 100%.

#### 2.4.2 Assessment of Anastomotic Patency

Anastomotic patency was assessed postoperatively at Weeks 1, 2, and 4 using repeat ICG lymphography, confirming the continued flow of ICG through the LVA site.

### 2.5 Statistical Analysis

All quantitative data are expressed as mean ± standard deviation (SD). Statistical analyses were performed using GraphPad Prism 9.0 (GraphPad Software, La Jolla, CA, USA). Comparisons of swelling rates among the five groups at specific time points were performed using a one‐way analysis of variance (ANOVA) followed by a Tukey's or Dunnett's post hoc test. A *p* value < 0.05 was considered statistically significant.

### 2.6 RNA Sequencing and Bioinformatics Analysis

Total RNA was extracted from lymphedematous tissue samples using the MJzol Animal RNA Isolation Kit (Majorbio, Shanghai, China) and purified with the RNAClean XP Kit (Beckman Coulter, USA) and RNase‐Free DNase Set (QIAGEN, Germany). RNA integrity was assessed using an Agilent 2100 Bioanalyzer (Agilent Technologies, USA), and RNA concentration and purity were measured using a Qubit 2.0 Fluorometer and NanoDrop ND‐2000 spectrophotometer (Thermo Fisher Scientific, USA).

Sequencing libraries were constructed using the Dual‐mode mRNA Library Prep Kit (Yeasen, Shanghai, China) following the manufacturer's protocol, which included mRNA isolation, fragmentation, first‐ and second‐strand cDNA synthesis, end repair, 3′ adenylation, adapter ligation, and PCR enrichment. Library quality was verified using an Agilent 4200 TapeStation. Paired‐end sequencing (PE150) was performed on the Illumina NovaSeq 6000 platform.

Raw reads were preprocessed using Seqtk (v1.0) to remove adapter sequences, low‐quality bases (*Q* < 20), and short reads (< 25 bp). Ribosomal RNA reads were removed using Bowtie2 (v2.2.6). Clean reads were aligned to the rabbit reference genome (OryCun2.0) using HISAT2 (v2.0.4) with default parameters. Gene‐level expression was quantified using StringTie (v1.3.3b) and normalized by the TMM (trimmed mean of *M* values) method. Differential gene expression analysis was performed using edgeR (v3.2.0), with significance thresholds set at |log_2_ fold change| ≥ 1 and adjusted *Q*‐value ≤ 0.05. Functional enrichment analysis of differentially expressed genes was conducted using Gene Ontology (GO) and Kyoto Encyclopedia of Genes and Genomes (KEGG) pathway databases.

## 3 Results

### 3.1 Successful Establishment and Validation of a Chronic Lymphedema Model

All 25 rabbits exhibited comparable baseline body weight and hind limb circumference prior to surgery. Postoperatively, all animals survived with stable vital signs throughout the observation period. Hind limb blood perfusion was well maintained, and no instances of limb necrosis, obvious claudication, or severe distress were observed, confirming the safety and feasibility of the surgical protocol.

Macroscopically, animals in the untreated secondary lymphedema (SLD) group developed progressive limb edema. The limb swelling rate (SR) increased rapidly from a baseline of 0.3% ± 0.9% to 24.9% ± 2.5% at Week 4 post‐surgery. Swelling peaked at 28.5% ± 4.1% by Week 8 and maintained a stable plateau until the 16‐week endpoint (Figure 3).

**FIGURE 3:**
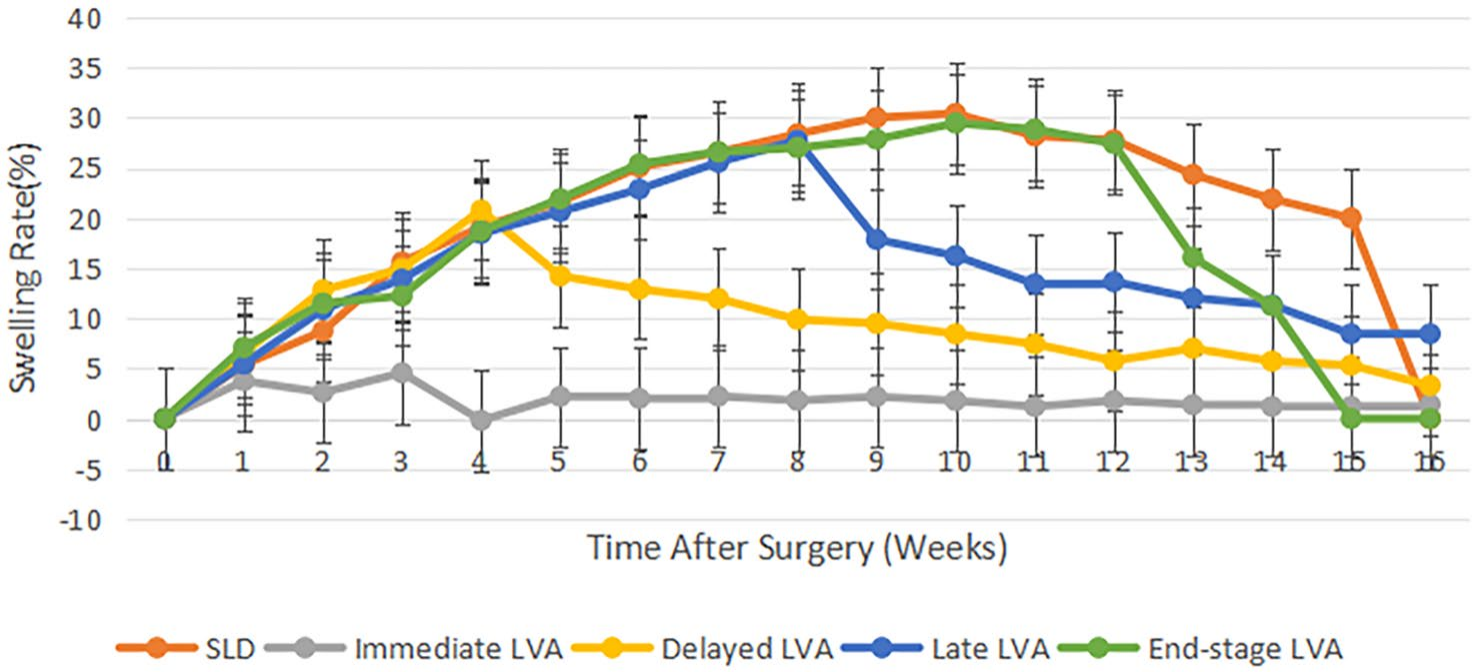
Time‐course evaluation of hind limb swelling rates across different groups. The immediate LVA group demonstrated stable and minimal swelling throughout the observation period, whereas the untreated SLD group showed progressive limb swelling that plateaued at approximately 30% around Weeks 9–10 before declining sharply after Week 15. LVA performed at delayed, late, and end‐stage disease timepoints (Delayed LVA, Late LVA, and End‐stage LVA groups) resulted in swelling trajectories that closely paralleled the SLD group during the early phase but diverged progressively thereafter: the Delayed LVA group showed the earliest and most sustained reduction in swelling, followed by the Late LVA group, while the End‐stage LVA group showed the latest onset of improvement, converging toward baseline only after Week 12–13. These results indicate that earlier surgical intervention is associated with more rapid and durable resolution of lymphedema. Data are presented as mean ± SD.

To further validate the model at the molecular level, tissue analysis at 4 weeks post‐surgery demonstrated a significant upregulation of key lymphangiogenic markers. Compared to the control group, the expression of VEGF‐C was upregulated by 2.45‐fold (*p* = 0.012), and LYVE‐1 was upregulated by 2.74‐fold (*p* = 0.010). These combined macroscopic and transcriptomic alterations confirmed the successful establishment of a stable, irreversible chronic lymphedema model.

Macroscopic and histopathological evaluations further corroborated the progressive structural deterioration of the lymphatic system. Compared to the normal popliteal lymph node architecture (Figure 4A), the extirpated tissues from the SLD group exhibited severe structural disruption and macroscopic tissue edema (Figure 4B). High‐magnification histological analysis revealed extensive extracellular matrix deposition and marked tissue fibrosis (Figure 4C). Furthermore, severe lymphocyte depletion and widespread infiltration of chronic inflammatory cells, predominantly macrophages, were observed within the residual lymphoid architecture (Figure 4D), confirming the irreversible pathological transition in this model.

**FIGURE 4:**
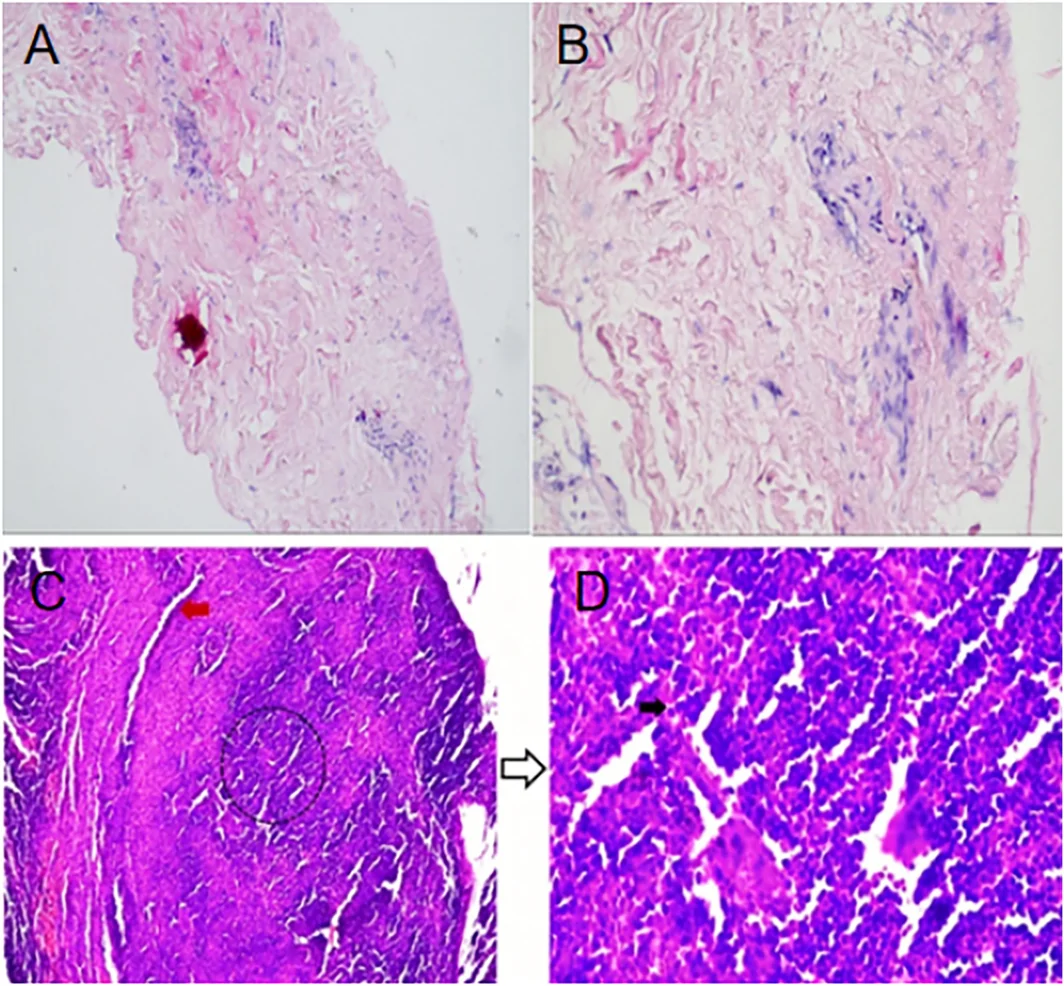
Histopathological evaluation of popliteal lymphatic tissues. (A) Normal architecture of a healthy popliteal lymph node in the control group. (B) Macroscopic tissue edema and severe structural disruption of the residual lymphatic tissues in the untreated SLD group at 4 weeks post‐surgery. (C) High‐magnification view showing extensive extracellular matrix deposition and marked tissue fibrosis in the SLD group. (D) Widespread infiltration of chronic inflammatory cells (predominantly macrophages) and severe lymphocyte depletion observed in the fibrotic lymphatic architecture of the SLD group. Black arrow indicates the residual lymphoid architecture within the fibrotic popliteal lymphatic tissue.

### 3.2 LVA Alleviates Limb Edema and Restores Lymphatic Drainage in a Time‐Dependent Manner

The technical feasibility and success of the LVA procedure were confirmed intraoperatively. The average diameter of the targeted lymphatic vessels was 0.6 ± 0.2 mm, and the adjacent veins measured 0.8 ± 0.3 mm. The mean time from incision to the completion of the anastomosis was 55.3 ± 15.3 min. Serial ICG lymphography demonstrated that early intervention significantly favored long‐term anastomotic function. In the immediate LVA group, 100% (5/5) of the rabbits maintained patent anastomoses throughout the 16‐week observation period. In the delayed intervention cohort, the procedural completion rate was 93.3% (14/15, with one exclusion due to self‐inflicted wound dehiscence). At the study endpoint, the patency rates in the delayed groups were notably lower: 75% (3/4) in the 4‐week LVA group, 80% (4/5) in the 8‐week LVA group, and 80% (4/5) in the 12‐week LVA group.

Consistent with the patency results, the therapeutic effect of LVA on macroscopic limb edema was strictly dependent on intervention timing (Figure 3). Rabbits receiving immediate LVA exhibited a powerful preventive effect, showing only a transient and minimal increase in limb swelling that peaked at just 5.5% ± 2.8% before stabilizing.

In contrast, delayed LVA performed on already established lymphedema yielded partial but significant therapeutic responses. Following the procedure, the 4‐week LVA group showed a substantial decrease in swelling rate from a preoperative high of 25.1% ± 3.7% down to 10.5% ± 2.3% by the study endpoint. Similarly, the 8‐week LVA group experienced a reduction from 28.3% ± 4.1% down to 14.3% ± 3.0%.

At the final 16‐week evaluation, while all LVA‐treated groups exhibited significantly lower swelling rates compared to the untreated SLD group (28.7% ± 4.1%, *p* < 0.05), the immediate LVA group (5.5% ± 2.8%) demonstrated significantly superior long‐term outcomes compared to both the 4‐ and 8‐week delayed intervention groups (*p* < 0.05). These data are consistent with the fundamental principle that early surgical intervention maximizes both anastomotic survival and therapeutic efficacy.

### 3.3 Transcriptomic Profiling Defines the Pathological Landscape of SLD

To fully elucidate the molecular mechanisms underlying secondary lymphedema, we performed high‐throughput transcriptomic sequencing on tissues from the untreated SLD model and the Control group. The volcano plot (Figure S3A) visually presents the significant gene expression alterations induced by the disease.

Compared to the Control group, a substantial number of genes were differentially expressed in the SLD model. The top 20 significantly upregulated genes (highlighted in red) were predominantly enriched in pathways associated with inflammatory responses, immune cell chemotaxis, extracellular matrix (ECM) remodeling, and tissue fibrosis. Notably, the expression of Wnt signaling pathway inhibitors, such as WIF1 (Fold Change = 158.3) and SFRP2 (Fold Change = 98.5), increased dramatically, suggesting a critical role of Wnt signaling in lymphedema‐associated fibrosis. Furthermore, several key pro‐fibrotic molecules, including MMP9, COL1A1, POSTN, and FN1, were markedly upregulated.

In stark contrast, the top 20 significantly downregulated genes (highlighted in blue) were highly concentrated in biological processes related to adipocyte differentiation, lipid metabolism, and the maintenance of muscle tissue homeostasis. Key regulators of adipose tissue, such as fatty acid‐binding protein 4 (FABP4, FC = −112.4) and adiponectin (ADIPOQ, FC = −98.7), exhibited precipitous declines in expression. These findings clearly delineate a molecular signature of SLD characterized by the robust activation of pro‐fibrotic/inflammatory signals and the progressive suppression of normal metabolic functions, particularly lipid metabolism.

### 3.4 Immediate LVA Reverses the Pathological Gene Expression Signature of SLD

To investigate the crucial impact of intervention timing on therapeutic outcomes at the molecular level, we directly compared the transcriptomic profiles of the “Immediate LVA” and “Late LVA” (8‐week) groups. The results (Figure S3B) were striking, demonstrating the profound capacity of immediate LVA to reverse the pathological progression.

The volcano plot reveals a “mirror effect” when comparing Immediate LVA to Late LVA. In the Immediate LVA group, genes that exhibited significantly higher expression levels (highlighted in green) were precisely those functional genes suppressed in the SLD model. For instance, the expression levels of FABP4 (FC = 95.3) and ADIPOQ (FC = 81.2) were effectively rescued, indicating that early LVA prevents the deterioration of lipid metabolism and promotes the restoration of adipose tissue function.

Conversely, genes that were significantly suppressed in the Immediate LVA group (highlighted in purple) were the core pathogenic genes activated in the SLD model. Key drivers of fibrosis and inflammation, including WIF1 (FC = −120.1), SFRP2 (FC = −85.4), and FAP (FC = −71.2), were strongly inhibited. These findings suggest that immediate LVA is associated with suppression of the inflammation‐fibrosis gene signature at the transcriptomic level, whereas delayed intervention (Late LVA) shows a less complete molecular reversal, consistent with the clinical principle of “early intervention, early benefit” for SLD.

### 3.5 Heatmap Analysis Reveals a Time‐Dependent Loss of Transcriptomic Homeostasis

To visualize the global transcriptomic dynamics and evaluate the efficacy of LVA intervention at different time points, we constructed a hierarchical clustering heatmap of key differentially expressed genes across all six experimental groups (Figure S4). The heatmap provides a compelling visual representation of how the timing of surgical intervention dictates the molecular trajectory of the tissue.

The most striking observation is the stark contrast between the two ends of the timeline. The healthy control group is characterized by a nearly uniform suppression of these targeted genes (represented by solid blue). Crucially, the immediate LVA group almost perfectly replicates this healthy transcriptomic signature. The overarching blue pattern in this column indicates that immediate surgical intervention successfully prevents the activation of the pathological gene program, effectively maintaining tissue homeostasis.

In contrast, the untreated SLD only group exhibits a massive upregulation of a large cluster of genes (prominently displayed as bright red in the upper section of the heatmap), which includes key fibrotic and inflammatory markers such as COL1A1, KRT10, and WIF1.

By tracking the intermediate groups, a clear temporal gradient of transcriptomic deterioration emerges:

1. 4‐week LVA: The pure blue signature begins to break down, showing a transition to pale blue and white, indicating early signs of gene activation and the initial loss of molecular stability.
2. 8‐week LVA: The dysregulation becomes more pronounced, with the emergence of distinct red clusters in the lower half of the heatmap, suggesting an aberrant remodeling process as the tissue attempts to compensate for prolonged lymphatic stasis.
3. 12‐week LVA: The upper gene cluster undergoes a dramatic shift to intense red, heavily mirroring the pathological expression profile of the untreated SLD only group.

Taken together, these heatmap patterns suggest that delayed LVA intervention may not be sufficient to fully halt the progression of lymphedema‐associated transcriptional changes. The progressive shift from a “healthy blue” to a “pathological red” signature strongly underscores the critical importance of early, preferably immediate, surgical intervention to preserve physiological gene expression and prevent irreversible fibrotic remodeling.

## 4 Discussion

Secondary lymphedema, a prevalent and debilitating complication following oncological lymph node dissection, remains a significant clinical challenge due to the progressive nature of lymphatic fibrosis and adipose tissue deposition [21, 22]. While lymphaticovenous anastomosis (LVA) has emerged as a frontline physiological treatment, its efficacy is profoundly influenced by the timing of intervention [7, 8]. The core challenge lies in the pathological transition from a fluid‐dominant, reversible state to a solid‐dominant, irreversible state, which compromises the integrity of remaining lymphatic vessels and reduces the likelihood of successful surgical outcomes [23, 24]. This study successfully establishes and validates a novel, stable rabbit model of irreversible lymphedema, providing a robust platform to systematically investigate the time‐dependent efficacy of LVA and its underlying molecular mechanisms.

The present rabbit model was developed in dialogue with existing literature. A prior study demonstrated the technical feasibility of LVA in rabbits as a supermicrosurgical training tool [25], but did not address edema induction or time‐dependent outcomes. Subsequent models employing popliteal lymph node excision alone produced lymphatic dysfunction detectable by ICG lymphography but did not consistently result in sustained macroscopic limb swelling [19, 20]. The present study resolved this limitation by combining lymph node excision with circumferential skin incision, which reliably disrupts collateral drainage pathways and produces reproducible hind limb edema, enabling systematic investigation of LVA timing.

Our findings suggest that immediate LVA is associated with markedly reduced development of chronic lymphedema, as evidenced by near‐complete prevention of sustained limb swelling in this rabbit model. This aligns with clinical observations suggesting that early or prophylactic LVA in high‐risk patients yields superior long‐term results by intervening before the vicious cycle of lymphatic stasis, inflammation, and fibrosis is fully established [9, 26]. Furthermore, recent clinical evidence supports the notion that earlier intervention yields superior outcomes: a 2024 multicenter randomized controlled trial (N‐LVA) demonstrated improvements in physical and mental function domains following LVA, and prophylactic approaches such as the LYMPHA procedure—performed at the time of axillary lymph node dissection—have shown particular promise in preventing lymphedema onset altogether [27]. This aligns with our finding that immediate LVA in the rabbit model achieved superior and sustained control of limb swelling compared with delayed intervention, reinforcing the clinical relevance of early surgical timing. In our model, the 100% patency rate in the immediate group underscores the technical advantage of operating on healthy, non‐fibrotic lymphatic vessels. In contrast, while delayed LVA on established lymphedema still offered significant therapeutic benefits, its efficacy was diminished. The observed decrease in anastomotic patency and the incomplete reduction in limb volume in the delayed groups (4, 8, and 12 weeks) are consistent with clinical reports where LVA in advanced stages provides palliation rather than a complete cure [28]. This time‐dependent decline in efficacy highlights a critical “therapeutic window,” after which pathological changes likely become too severe for complete reversal.

A key strength of this study is the integration of transcriptomic analysis to dissect the molecular landscape of lymphedema progression and the response to LVA. The upregulation of pro‐inflammatory and pro‐fibrotic pathways, including TNF, IL‐17, and TGF‐β signaling, in the untreated lymphedema group provides a molecular blueprint for the tissue remodeling that characterizes chronic disease [29, 30]. These pathways are well‐established drivers of fibroblast activation, collagen deposition, and ultimately, tissue fibrosis, which clinically manifests as irreversible limb hardening [31].

Crucially, immediate LVA was associated with significant downregulation of these inflammatory and fibrotic signaling cascades, suggesting that early intervention may attenuate the molecular processes driving disease progression. This molecular rescue explains the superior clinical outcomes observed in the immediate group. The significant downregulation of genes associated with immune cell infiltration and activation (e.g., via Chemokine and PI3K‐Akt signaling pathways) suggests that restoring lymphatic outflow promptly may help limit the chronic inflammatory state that perpetuates tissue damage [32]. Conversely, delayed LVA could only partially mitigate these molecular changes, which correlates with the less complete clinical improvement. These transcriptomic findings are consistent with a model in which early LVA helps limit inflammation‐driven fibrosis, although this association does not establish direct causality.

Furthermore, our analysis of the HIF‐1 signaling pathway offers novel insights into the role of tissue hypoxia in lymphedema pathogenesis. The upregulation of HIF‐1α in the untreated group suggests that chronic lymphatic stasis induces a hypoxic microenvironment, which is known to be a powerful stimulus for both fibrosis and pathological lymphangiogenesis [33, 34]. The downregulation of HIF‐1 pathway genes following immediate LVA is consistent with improved fluid homeostasis, suggesting that early intervention may attenuate hypoxia‐driven disease progression. This finding opens potential avenues for novel adjuvant therapies aimed at modulating hypoxic responses in lymphedema treatment.

While this rabbit model successfully recapitulates key features of human secondary lymphedema, certain limitations must be acknowledged. The acute, surgically‐induced nature of lymphedema in this model may not fully replicate the more gradual onset sometimes seen in clinical practice. Additionally, while our 16‐week follow‐up period confirmed the establishment of chronic changes, longer term studies could provide further insights into the durability of LVA outcomes. The relatively small sample size (*n* = 5 per group) is a limitation of this study; however, consistent and reproducible results across all animals support the validity of our findings. Future studies with larger cohorts are warranted to confirm these results. Additionally, the transcriptomic findings, while informative, were not independently validated by complementary methods such as quantitative PCR, Western blotting, or immunohistochemistry; future studies incorporating such validation would further strengthen confidence in the identified molecular pathways. This study was conducted exclusively in male rabbits; generalizability to female subjects, who represent the predominant clinical population for secondary lymphedema, warrants further investigation. Nonetheless, this work provides a validated, clinically relevant large‐animal model for future preclinical studies, including the evaluation of novel anti‐inflammatory or anti‐fibrotic pharmacological agents as adjuncts to LVA surgery [35, 36].

## 5 Conclusion

In conclusion, this study provides multilevel evidence from a rabbit model that the timing of LVA is a critical determinant of therapeutic success. Immediate LVA was associated with prevention of chronic lymphedema development, as evidenced by maintained anastomotic patency and attenuation of transcriptomic signatures consistent with inflammation and fibrosis. While delayed LVA offers significant palliation, its efficacy is progressively compromised as the disease advances. These findings underscore the importance of early diagnosis and intervention in the management of secondary lymphedema and establish a valuable preclinical platform for optimizing future treatment strategies.

## Author Contributions

Ping Zhao conceptualized the study, designed the specific experimental protocols, performed the majority of the experiments, acquired and analyzed the data, and wrote the original draft of the manuscript. Xun Li provided overall conceptualization, contributed to the study design, supervised the project, and critically reviewed and edited the manuscript. Wenzhao Liu, Mingqiang Liu, and Jintao Yang performed experiments and contributed to data acquisition. Wenzhao Liu, Sen Li, and Jintao Yang were involved in data analysis. All authors have read and approved the final manuscript.

## Funding

The authors have nothing to report.

## Ethics Statement

All animal experiments and procedures were approved by the Institutional Animal Care and Use Committee of The First Hospital of Lanzhou University (Approval number: LDYYLL2026‐218). The care and use of the animals were conducted in full compliance with local, national, ethical, and regulatory principles, and the study was reported in accordance with the ARRIVE guidelines.

## Conflicts of Interest

The authors declare no conflicts of interest.

## Supporting information


**Figure S1:** Preoperative Indocyanine Green (ICG) lymphography mapping. Real‐time fluorescence imaging reveals the physiological lymphatic drainage pathways in the rabbit right hind limb prior to surgical intervention. ICG was injected subcutaneously at the interdigital web spaces.
**Figure S2:** Surgical establishment of the secondary lymphedema (SLD) model. Intraoperative photographs showing the identification and complete excision of the popliteal lymph nodes, followed by ligation of the afferent lymphatic vessels to induce sustained lymphatic obstruction.
**Figure S3:** Volcano plots of differentially expressed genes. (A) Transcriptomic comparison between the untreated SLD group and the normal Control group. Red dots represent significantly upregulated genes (e.g., pro‐fibrotic markers like WIF1, SFRP2, COL1A1), and blue dots represent significantly downregulated genes (e.g., lipid metabolism regulators like FABP4, ADIPOQ). (B) Transcriptomic comparison highlighting the “mirror effect” between the Immediate LVA group and the Late LVA (8‐week) group. Immediate LVA effectively rescues the expression of metabolic genes (green dots) and powerfully suppresses the activation of pro‐fibrotic pathogenic genes (purple dots).
**Figure S4:** Hierarchical clustering heatmap of transcriptomic profiling across different intervention timelines. The heatmap visually demonstrates a time‐dependent loss of transcriptomic homeostasis. The Immediate LVA group nearly completely replicates the “healthy blue” gene expression signature of the normal Control group. In contrast, progressive delays in LVA intervention (4‐, 8‐, and 12‐week LVA groups) result in a gradual transition toward the “pathological red” signature characteristic of the untreated SLD group, emphasizing the necessity of early intervention to prevent irreversible fibrotic remodeling.

## Data Availability

The raw data supporting the findings of this study have been archived in the institutional scientific research data archiving system (https://www.lzdxdyyy.com/Web/SpecialTopic/Detail/5153f91b‐923e‐c04a‐5060‐3a21c8165e84?rootTypeId=121) in accordance with the data management policy of Lanzhou University. As this is an internal institutional repository intended primarily for research integrity oversight, the data are not openly accessible to the public; however, they are available from the corresponding author upon reasonable request for the purpose of scientific verification.
