# Supplementary material for: Time‐Dependent Efficacy of Lymphaticovenous Anastomosis in Secondary Lymphedema: A Rabbit Model Study
Source: FASEB J. 2026 Oct 5;40(19):e72327. doi: 10.1096/fj.202602801RR (PMC13635956; doi:10.1096/fj.202602801RR)
Supplement: Supplementary file 1 [file FSB2-40-e72327-s001.docx]

****Supplementary Figure S1. Preoperative Indocyanine Green (ICG) lymphography mapping.****
Real-time fluorescence imaging reveals the physiological lymphatic drainage pathways in the rabbit right hind limb prior to surgical intervention. ICG was injected subcutaneously at the interdigital web spaces.

****Supplementary Figure S2. Surgical establishment of the secondary lymphedema (SLD) model.****
Intraoperative photographs showing the identification and complete excision of the popliteal lymph nodes, followed by ligation of the afferent lymphatic vessels to induce sustained lymphatic obstruction.

****Supplementary Figure S3. Volcano plots of differentially expressed genes.****
****(A)**** Transcriptomic comparison between the untreated SLD group and the normal Control group. Red dots represent significantly upregulated genes (e.g., pro-fibrotic markers like WIF1, SFRP2, COL1A1), and blue dots represent significantly downregulated genes (e.g., lipid metabolism regulators like FABP4, ADIPOQ). ****(B)**** Transcriptomic comparison highlighting the "mirror effect" between the Immediate LVA group and the Late LVA (8-week) group. Immediate LVA effectively rescues the expression of metabolic genes (green dots) and powerfully suppresses the activation of pro-fibrotic pathogenic genes (purple dots).

****Supplementary Figure S4. Hierarchical clustering heatmap of transcriptomic profiling across different intervention timelines.****
The heatmap visually demonstrates a time-dependent loss of transcriptomic homeostasis. The Immediate LVA group nearly completely replicates the "healthy blue" gene expression signature of the normal Control group. In contrast, progressive delays in LVA intervention (4-week, 8-week, and 12-week LVA groups) result in a gradual transition toward the "pathological red" signature characteristic of the untreated SLD group, emphasizing the necessity of early intervention to prevent irreversible fibrotic remodeling.
